# p27^KIP1^ is abnormally expressed in Diffuse Large B-Cell Lymphomas and is associated with an adverse clinical outcome

**DOI:** 10.1038/sj.bjc.6690539

**Published:** 1999-07

**Authors:** Al Sáez, E Sánchez, M Sánchez-Beato, M A Cruz, I Chacón, E Muñoz, F I Camacho, J C Martínez-Montero, M Mollejo, J F Garcia, M A Piris

**Affiliations:** Departments of Pathology,Oncology and Preventive Medicine, Virgen de la Salud Hospital, Toledo, 45004, Spain

**Keywords:** p27^KIP1^, MIB1, CDK inhibitors, diffuse large B-cell lymphoma, survival analysis

## Abstract

Cell cycle progression is regulated by the combined action of cyclins, cyclin-dependent kinases (CDKs), and CDK-inhibitors (CDKi), which are negative cell cycle regulators. p27^KIP1^ is a CDKi key in cell cycle regulation, whose degradation is required for G1/S transition. In spite of the absence of p27^KIP1^ expression in proliferating lymphocytes, some aggressive B-cell lymphomas have been reported to show an anomalous p27^KIP1^ staining. We analysed p27^KIP1^ expression in a series of Diffuse Large B-cell Lymphoma (DLBCL), correlating it with the proliferative index and clinical outcome, to characterize the implications of this anomalous staining in lymphomagenesis in greater depth. For the above mentioned purposes, an immunohistochemical technique in paraffin-embedded tissues was employed, using commercially available antibodies, in a series of 133 patients with known clinical outcomes. Statistical analysis was performed in order to ascertain which clinical and molecular variables may influence outcome, in terms of disease-free survival (DFS) and overall survival (OS). The relationships between p27^KIP1^ and MIB-1 (Ki-67) were also tested. An abnormally high expression of p27^KIP1^ was found in lymphomas of this type. The overall correlation between p27^KIP1^ and MIB-1 showed there to be no significant relationship between these two parameters, this differing from observations in reactive lymphoid and other tissues. Analysis of the clinical relevance of these findings showed that a high level of p27^KIP1^ expression in this type of tumour is an adverse prognostic marker, in both univariate and multivariate analysis. These results show that there is abnormal p27^KIP1^ expression in DLBCL, with adverse clinical significance, suggesting that this anomalous p27^KIP1^ protein may be rendered non-functional through interaction with other cell cycle regulator proteins. © 1999 Cancer Research Campaign


*British Journal of Cancer* (1999) **80**(9), 1427–1434
© 1999 Cancer Research Campaign
Article no. bjoc.1999.0539

### p27KIP1 is abnormally expressed in Diffuse Large B-Cell Lymphomas and is associated with an adverse clinical outcome

**AI Sáez, E Sánchez, M Sánchez-Beato, MA Cruz, I Chacón, E Muñoz, FI Camacho, JC Martínez-Montero, M Mollejo, JF García and MA Piris**

Departments of Pathology, Oncology and Preventive Medicine, Virgen de la Salud Hospital, Toledo 45004, Spain

**Summary** Cell cycle progression is regulated by the combined action of cyclins, cyclin-dependent kinases (CDKs), and CDK-inhibitors (CDKi), which are negative cell cycle regulators. $p27^{KIP1}$ is a CDKi key in cell cycle regulation, whose degradation is required for G1/S transition. In spite of the absence of $p27^{KIP1}$ expression in proliferating lymphocytes, some aggressive B-cell lymphomas have been reported to show an anomalous $p27^{KIP1}$ staining. We analysed $p27^{KIP1}$ expression in a series of Diffuse Large B-cell Lymphoma (DLBCL), correlating it with the proliferative index and clinical outcome, to characterize the implications of this anomalous staining in lymphomagenesis in greater depth. For the above mentioned purposes, an immunohistochemical technique in paraffin-embedded tissues was employed, using commercially available antibodies, in a series of 133 patients with known clinical outcomes. Statistical analysis was performed in order to ascertain which clinical and molecular variables may influence outcome, in terms of disease-free survival (DFS) and overall survival (OS). The relationships between $p27^{KIP1}$ and MIB-1 (Ki-67) were also tested. An abnormally high expression of $p27^{KIP1}$ was found in lymphomas of this type. The overall correlation between $p27^{KIP1}$ and MIB-1 showed there to be no significant relationship between these two parameters, this differing from observations in reactive lymphoid and other tissues. Analysis of the clinical relevance of these findings showed that a high level of $p27^{KIP1}$ expression in this type of tumour is an adverse prognostic marker, in both univariate and multivariate analysis. These results show that there is abnormal $p27^{KIP1}$ expression in DLBCL, with adverse clinical significance, suggesting that this anomalous $p27^{KIP1}$ protein may be rendered non-functional through interaction with other cell cycle regulator proteins.

**Keywords:** $p27^{KIP1}$; MIB1; CDK inhibitors; diffuse large B-cell lymphoma; survival analysis

Cell cycle progression is promoted by the action of the cyclin-dependent kinases (CDKs) and their activating sub-units, the cyclins. The G1/S transition in mammalian cells is believed to be regulated by complexes of Cyclin E/CDK2 and Cyclin D/CDK4 or CDK6; the kinase activity of these CDKs requires an association between a catalytic sub-unit, the CDK, and a regulatory sub-unit, the cyclin. This kinase activity can be inhibited by various CDK inhibitors (CDKis). There are two main groups of CDKis, the CIP1/KIP1 ($p21^{WAF1/CIP1}$ (El-Deiry et al, 1993; Harper et al, 1993), $p27^{KIP1}$ (Polyak et al, 1994; Toyoshima and Hunter, 1994) and $p57^{KIP2}$ (Lee et al, 1995; Matsuoka et al, 1995) and the INK4 ($p16^{INK4A}$, $p15^{INK4B}$, $p18^{INK4C}$ and $p19^{INK4D}$ (Serrano et al, 1993; Guan et al, 1994; Hannon and Beach, 1994; Chan et al, 1995)) families. Members of the CIP1/KIP1 family share sequence homology, and are able to inhibit a broader range of cyclin–CDKs. Nevertheless, members of the INK4 family mainly inhibit the kinase activity of CDK4-CDK6. All of these CDKis cause G1 arrest when overexpressed in transfected cells.

$p27^{KIP1}$ protein inhibits CDK activity by mediating cell cycle arrest (Polyak et al, 1994; Toyoshima and Hunter, 1994). In vitro, $p27^{KIP1}$ has been found to be involved in G1 arrest in response to extracellular signals such as transforming growth factor (TGF)-β in epithelial cells (Polyak et al, 1994), cell–cell contact in fibroblasts (Fang et al, 1995), cAMP in murine macrophages (Kato et al, 1994) and rapamycin in T-lymphocytes (Nourse et al, 1994), all of which are agents known to inhibit cell cycle progression. $p27^{KIP1}$ has been implicated as the CDKi involved in mitogen-dependent CDK regulation, as a key protein in passage through the restriction point (Couts et al, 1996), thus making the $p27^{KIP1}$ degradation a necessary step in the irreversible decision to enter the S phase (Cheng et al, 1998). $p27^{KIP1}$ protein is present in quiescent cells, and levels descend dramatically when cells are stimulated by growth factors (Kato et al, 1994; Nourse et al, 1994). Besides this key role in cell cycle control, additional functions for $p27^{KIP1}$ in cell differentiation (Hengst and Reed, 1996), apoptosis induction (Bani et al, 1995; Wang et al, 1997) and chemotherapy resistance (Croix et al, 1996) have been proposed. Knockout mice for $p27^{KIP1}$ show an increase in the size of organs and the cell number, confirming its inhibitory role in cell cycle control, in the same regulatory pathway as the gene Retinoblastoma (Rb) (Fero et al, 1996; Kiyokawa et al, 1996; Nakayama et al, 1996).

According to this model, quiescent non-tumoural lymphocytes express high levels of $p27^{KIP1}$ protein, which is down-regulated following cell activation. Thus, proliferating lymphoid cells, in either peripheral blood or peripheral lymphoid organs, express low or undetectable levels of $p27^{KIP1}$ protein (Croix et al, 1996; Sánchez-Beato et al, 1997). In lymphomas, when all histological types are taken into consideration, this relationship between $p27^{KIP1}$ expression and proliferation is generally preserved, with an inverse expression for $p27^{KIP1}$ and MIB-1 proteins. Tumours with a low

*Received 22 July 1998*
*Revised 31 November 1998*
*Accepted 20 January 1999*

*Correspondence to:* MA Piris proliferative index were mostly positive, while tumours characterized by a higher growth fraction had low $p27^{KIP1}$ protein levels (Sánchez-Beato et al, 1997), although in an earlier study performed by this group some exceptions to this rule were found in the group of aggressive B-cell lymphomas, including Burkitt's lymphoma and diffuse large B-cell lymphoma (DLBCL). Only very rare genetic alterations have been found in the $p27^{KIP1}$ gene (Shiohara et al, 1994; Kawamata et al, 1995; Morosetti et al, 1995; Pietenpol et al, 1995; Watanabe et al, 1995), suggesting that if inactivation of this CDKi takes place in human tumours, it would be through a mechanism other than genetic mutation, unlike the INK4 family.

Initial studies performed on breast, lung and colorectal cancer and melanoma, coincide in indicating that a high level of $p27^{KIP1}$ expression is associated in these tumours with a lower degree of malignancy (Fredersdorff et al, 1997) or a better clinical outcome (Bani et al, 1997; Catzavelos et al, 1997; Esposito et al, 1997; Loda et al, 1997; Porter et al, 1997), in spite of the fact that in these tumours there is no clear relationship between $p27^{KIP1}$ expression and growth fraction. Further studies offer differing data showing that a high level of $p27^{KIP1}$ expression may be associated with a more aggressive course in breast cancer and B-cell lymphocytic leukaemia (B-CLL) (Sgambato et al, 1997; Vrhorac et al, 1998). The presence of an elevated level of $p27^{KIP1}$ protein expression in subsets of tumours with high proliferative indexes suggests that the abnormally expressed protein lacks the functional capacity to inhibit cell cycle progression (Fredersdorf et al, 1997; Sgambato et al, 1997; Quintanilla-Martinez et al, 1998; Yatabe et al, 1998).

DLBCLs form a heterogeneous group in terms of clinical presentation, histology, immunophenotype, genetic alterations, response to treatment and prognosis (Harris et al, 1994). Recent findings suggest that the overall outcome for patients with DLBCL is decided by a combination of different clinical and biological variables, the expression of some of these cell cycle regulator proteins being specifically associated with different clinical features of tumours. Overall analysis showed that an extended overall survival (OS) time is associated with low growth fraction, low expression of p53 and MDM2 proteins and high Rb expression. In contrast with this finding, higher disease-free survival (DFS) time is mainly associated with low bcl2 expression (Slymen et al, 1990; Yamanaka et al, 1992; Miller et al, 1994; Piris et al, 1994; Martin et al, 1995; Hermine et al, 1996; Hill et al, 1996; Kramer et al, 1996; Gascoyne et al, 1997; Grogan et al, 1988; Sánchez et al, 1998)

Earlier studies in non-Hodgkins lymphomas (NHLs) pointed to the presence of a small group of DLBCL characterized by an unusually high level of $p27^{KIP1}$ expression (Sánchez-Beato et al, 1997; Quintanilla-Martinez et al, 1998), in which the opposing relationship between $p27^{KIP1}$ and MIB1 expression seemed to be lost. The main purpose of this work was to analyse the expression of this protein in greater depth, and to determine the clinical relevance of $p27^{KIP1}$ expression in this lymphoma type. To this end the clinical characteristics of a series of 133 cases of DLBCL were analysed, while simultaneously studying the relative clinical relevance of MIB1 expression, which is a growth fraction marker.

### MATERIALS AND METHODS

### Tissue samples, patients and clinical variables

Tumour specimens from 133 DLBCLs were obtained from the routine files of the Virgen de la Salud Hospital, and diagnosed according to the criteria used in the REAL classification (Harris et al, 1994). Consecutively diagnosed patients from 1980 to 1995 were included in the study if their cases fulfilled the following conditions:

- The initial diagnosis of DLBCL had to be made on a representative biopsy obtained before treatment.
- Paraffin-embedded, formalin-fixed tissue blocks from the diagnostic biopsy had to be available for immunohistochemical studies.
- All patients had to be treated with the aim of achieving a cure.

Untreated patients were excluded. Treatment decisions were not based on molecular and/or immunohistochemical features. Patients were treated according to standard therapeutic protocols, including polychemotherapy (CHOP or third-generation regimes, depending on IPI and clinical stage) and radiotherapy or surgery for localized tumours. A combination of these treatments was used for certain patients.

Age, clinical stage, performance status, LDH and the number of extranodal sites of the disease were used, as determined by the International Prognostic Index (IPI). IPI was grouped as 0–1, 2, 3 and 4–5. Complete remission (CR) was defined as the absence of clinical and radiological evidence of disease for a minimum of 4 weeks. Other degrees of response were considered to represent treatment failure. Patients who died of causes unrelated to the disease were censored at the time of death. OS was measured as the interval between the beginning of treatment and the death of the patient or the date of the last follow-up revision. Patients known to have died after finishing the treatment due to causes unrelated to the lymphoma (one colorectal cancer diagnosed shortly after the diagnosis of lymphoma, one acute myocardial infarct and one elderly patient with Alzheimer's disease) were considered as censored for the purposes of survival analysis. DFS was estimated for patients having CR, by measuring the interval between the end of the treatment and relapse or death attributable to the tumour, or the date of the last follow-up revision in patients who had no relapse. All data were checked for validity and consistency.

### Antibodies

$p27^{KIP1}$ protein was detected with a monoclonal antibody (mAb) from Transduction Laboratories, K25020 (1:1000 dilution), generated against the full-length mouse KIP1 protein. This antibody exclusively recognizes a 27 kDa protein, as previously shown by Mantel et al (1996). Ki-67, a nuclear protein expressed in the G1, S and G2/M phases, was detected by the MIB1 mAb (Novocastra) (1:50 dilution). It was also used as the control of antigenic preservation and successful antigenic retrieval.

### Immunostaining techniques

All immunostaining techniques were performed in paraffin-embedded tissue. A previous step of heat-induced antigen retrieval technique was used for all four antibodies. This took the form of pressure cooker heating in a solution of 0.01 M sodium citrate prior to incubation with the antibody (Ab). Following incubation with the primary Ab, immunodetection was performed with biotinylated anti-mouse immunoglobulins, followed by peroxidase-labelled streptavidine (LSAB-DAKO) and with diaminobencidine chromogen as substrate. All immunostaining was performed using the Techmate 500 (DAKO) automatic immunostaining device.

Incubation omitting the specific Ab, as well as with unrelated Abs, was used as a control of the technique. Internal control

 *© 1999 Cancer Research Campaign*

provided by benign reactive T-cells, macrophages, epithelial or endothelial cells was used to evaluate p27KIP1 and MIB1.

### Quantitative studies

All scoring and interpretations of immunohistochemical results were made by two pathologists (JFG and AIS), without knowledge of the clinical outcome or other clinical variables of tumour markers.

High magnification fields were chosen for the evaluation of p27KIP1, focusing on tumoural areas, counting up to 200 cells, excluding small T-cells or other non-tumoural cells. All immunoreactive cells were considered positive. The percentage of p27KIP1-positive tumoural cells was expressed as a ratio of positive cells to 100 cells. A manual procedure of counting p27KIP1 cells was used, in order to exclude all non-tumoural sub-populations (most of them expressing p27KIP1), using the morphology of the cells as criterion. A cut-off of 5% of tumoural cells was used for p27KIP1 expression, since this threshold roughly separates tumours with null or isolated p27KIP1 expression from those characterized by an intermediate or high level of expression, as determined by semi-quantitative analysis. This threshold divides the series into groups of similar size (p27 ≤ 5%: 57.9% cases; p27 > 5%: 42.1% cases), and this and closer cut-off points were found to be statistically highly significant. Moreover, the cut-off points at 4 and 6% were statistically significant, although they had the disadvantage of being less easily reproducible.

The Quantitative Proliferation Index application of the CAS 200 was used for the analysis of MIB1, since this measure of the tumoural growth fraction has been shown to correlate significantly with the clinical outcome in DLBCL (Sanchez-Bezto et al, 1997). This program determines a value expressing the growth fraction of the tumour, related to the number of proliferating cells. Representative fields down to a minimum of 30 000 $\mu^2$ were selected (approximately five fields with 45× objective and 10× ocular lenses). Sectional analysis was carried out, focusing on tumoural areas. A cut-off point of 20% of positive cells was used, as a previous study showed that this point discriminated between groups presenting different clinical behaviour. Categories were therefore classified as equal to or less than 20%, or greater than 20% (Sánchez et al, 1998).

### Statistical study

### *The relationship between p27 KIP1 and the proliferative index*

The distributions of p27KIP1 and MIB-1 were compared in order to establish any possible associations, using the Spearman correlation coefficient for doubly ordered data, and Kruskal–Wallis test for single ordered data. Differences were considered significant if $P < 0.05$.

### *Survival: univariate analysis*

The clinical variables analysed for survival studies were those included in the IPI (measured as 0–1, 2, 3, 4–5), which are: age (≤ 60 vs > 60), gender (female vs male), clinical stage (I + II vs III + IV), LDH (normal vs > normal). Actuarial survival curves (OS and DFS) were plotted using the Kaplan and Meier method (Kaplan and Meier, 1958). The statistical significance of associations between individual variables, OS and DFS was determined by using the log-rank test (Mantel, 1966). Cox's proportional

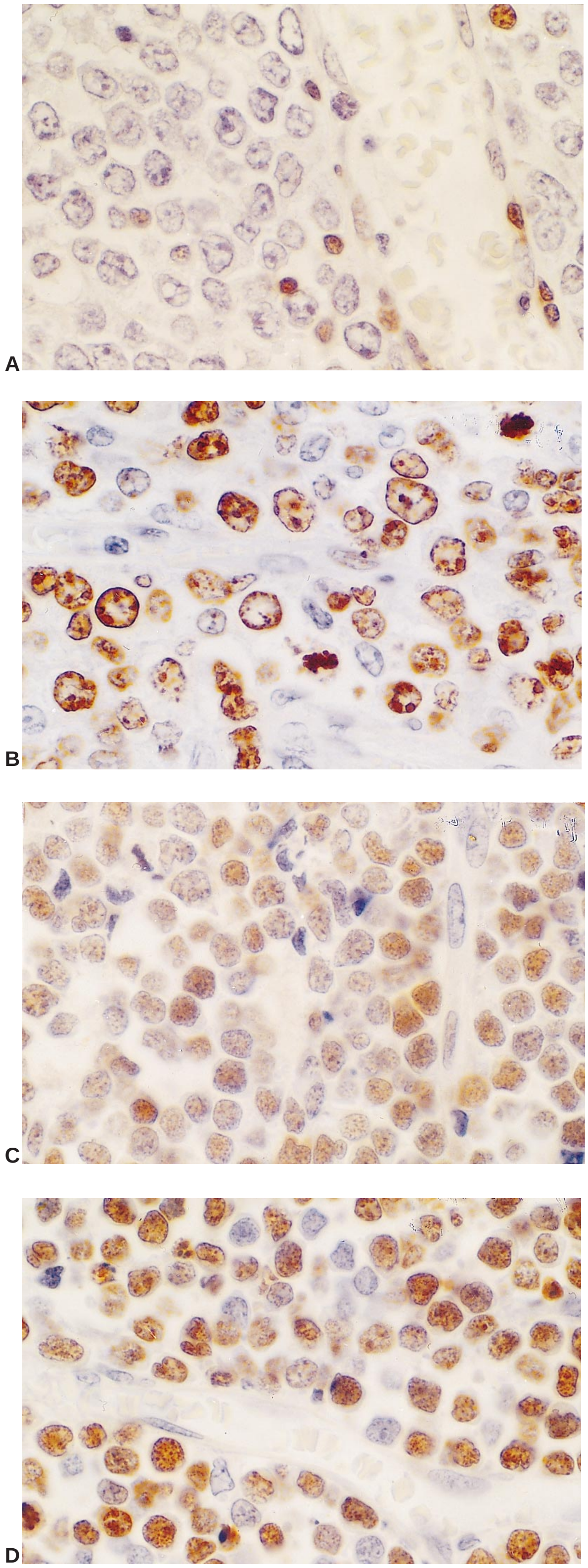

**Figure 1** Immunohistochemical expression of p27KIP1 and MIB1 in two different cases of the series. Case 65 was characterized by low p27KIP1 expression (1%), mainly restricted to small lymphocytes and endothelial cells (**A**), while most tumoural cells show strong MIB1 staining (**B**), read by the image analyser as 44.16%. Case 112 shows distinct p27KIP1 staining of a majority of tumoural cells (90%) (**C**) and relatively similar MIB1 staining (**D**) (43.94%)

*© 1999 Cancer Research Campaign* 

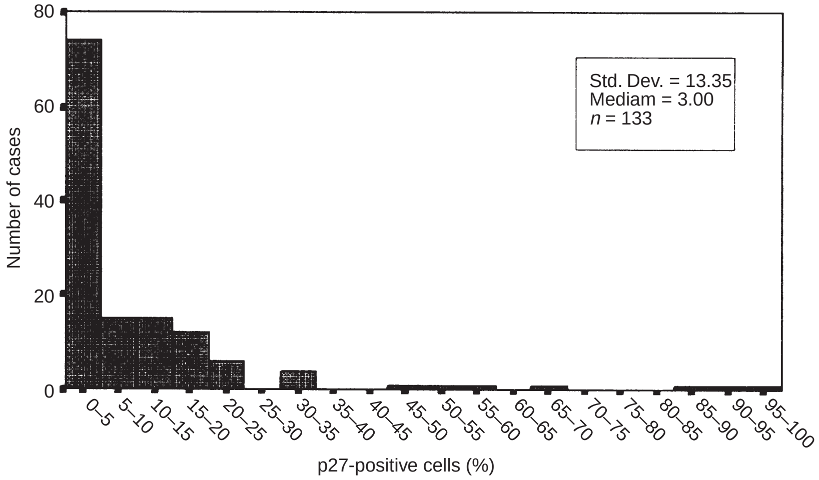

**Figure 2** Histogram of p27KIP1 expression in the DLBCL series

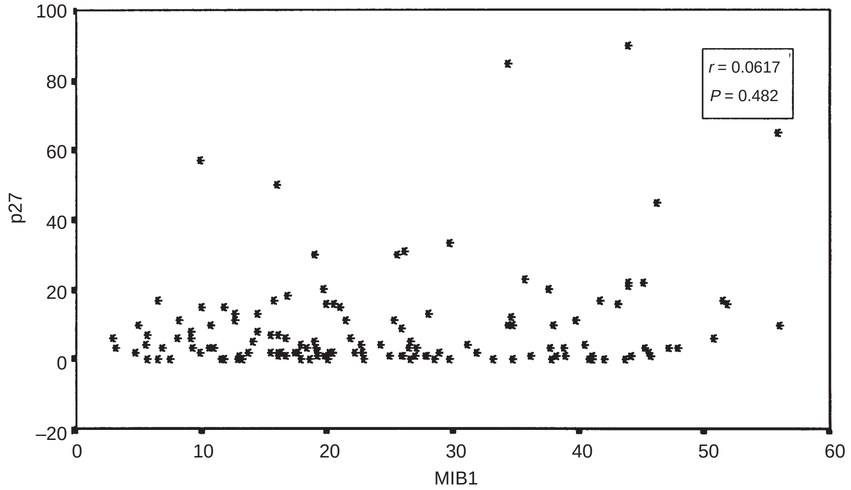

**Figure 3** Analysis of lineal correlation between p27KIP1 and MIB1. There is not a significant correlation between these two parameters hazard univariate analysis was also performed independently for each variable, calculating the relative risk (RR) of each variable in terms of survival and confidence interval (CI) (Cox, 1972).

### *Survival: multivariate analysis*

In order to identify the factors which might be of independent significance in influencing survival (OS and DFS), a Cox backward proportional hazard model was run (Cox, 1972). Variables included in the maximum models were: IPI (0–1, 2, 3 or 4–5), MIB1 (≤ 20% or > 20%), p27KIP1 (≤ 5% or > 5%). The lower values of IPI, MIB1 and p27KIP1, which had been found to be associated with longer survival probability, were used as reference levels (RL). All *P*-values were two-sided, and values of 0.05 or less were considered to indicate statistical significance.

The SPSS software package was used for all statistical analysis.

### RESULTS

### Characteristics of the patients

The clinical characteristics of the 133 patients included in this analysis have already been published (Sánchez et al, 1998).

### Immunohistochemical study p27KIP1 nuclear protein expression was observed in tumoural cells with a signal range of from 0.00 to 95.00 and with a median of 3.00. Null p27KIP1 staining was seen in 21 cases. p27KIP1 staining was found most frequently in a low to intermediate proportion of the tumoural cells. For survival analysis, a cut-off point of 5% of p27KIP1-positive cells was used, which divides the series into two groups, corresponding approximately to cases found by semi-quantitative analysis as negative or low-expression versus those with intermediate or high levels of expression (p27 ≤ 5%: 77 cases; p27 > 5%: 56 cases). A smaller group of seven cases (corresponding to percentile 95) showed a very high intensity of staining of most tumoural cells (Figure 1). A histogram of the overall distribution of p27KIP1 reactivity is shown in Figure 2.

MIB1 expression was observed in all cases, with a positive percentage of cell staining varying from 2.912 to 55.94 (median: 22.030) (Figure 1). In 73 cases (54.9%) the growth fraction was greater than 20%, this threshold having been found to be clinically relevant in previous studies (Sánchez et al, 1998). No significant correlation was found between p27KIP1 and MIB-1 expression,

© 1999 Cancer Research Campaign

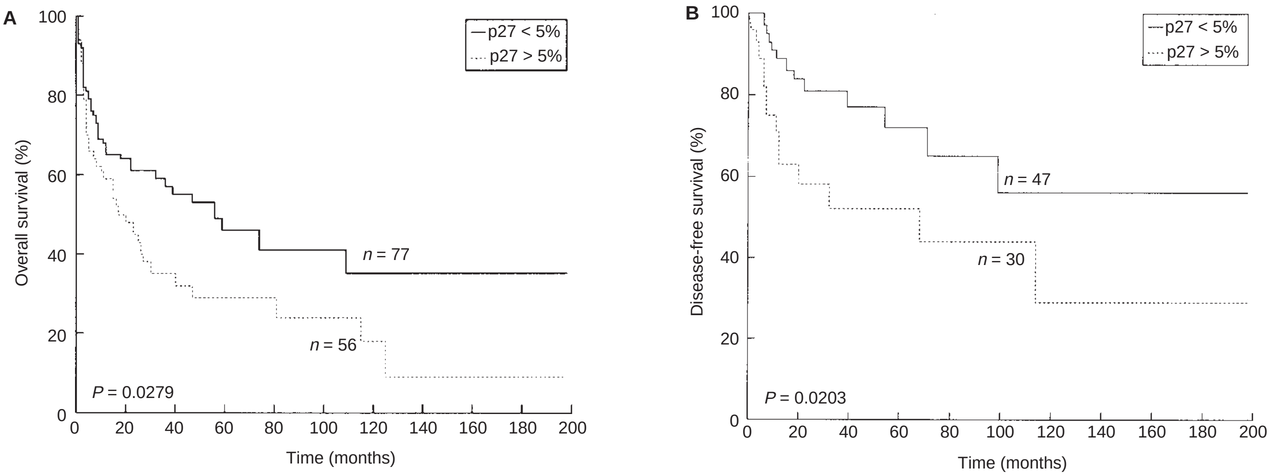

**Figure 4** Overall survival (**A**) and disease-free survival (**B**). Kaplan–Meier analysis of the series according to $p27^{KIP1}$ expression. Both OS and DFS were found to be significantly shortened in cases of DLBCL with higher $p27^{KIP1}$ expression using the Spearman correlation coefficient ($r = 0.0617$, $P = 0.482$) or the Kruskal–Wallis test ($\chi^2 = 0.0741$, $P = 0.7854$) (Figure 3).

### Survival analysis

Univariate and multivariate survival analyses were performed to analyse the prognostic significance of $p27^{KIP1}$ expression. MIB1 expression and the clinical parameters included in the IPI were included as references.

### *Univariate study*

Investigation of OS by Cox's analysis showed that high IPI ($P = 0.0004$), MIB1 > 20% ($P = 0.0199$) and $p27^{KIP1}$ > 5% ($P = 0.0320$) were significantly associated with shorter OS. The study of DFS showed that a high expression of $p27^{KIP1}$ ($P = 0.0412$) is significantly associated with shorter DFS. No significant relationship was found for high MIB1 or IPI (Table 1). Figure 4A shows the plots for OS, while Figure 4B shows the plot for DFS by Kaplan–Meier analysis and log-rank test.

### *Multivariate study*

Only low $p27^{KIP1}$ expression was found to be a parameter associated with prolonged OS ($P = 0.0145$), together with the clinical variables included within the IPI ($P = 0.0010$). The significance of MIB1, as found in univariate analysis, was absent from this series. When analysing DFS, only a high $p27^{KIP1}$ value was found to be significantly associated with a shorter DFS time ($P = 0.0424$) (Table 1).

### DISCUSSION

This study confirms that there is a group of DLBCL characterized by an anomalous $p27^{KIP1}$ expression, showing that in the DLBCL group as a whole the inverse relationship found in normal lymphoid

**Table 1** Univariate and multivariate analysis of the prognostic significance of $p27^{KIP1}$ and MIB1 expression in relation with the clinical parameters included within the IPI (Cox's regression model)

| | | Univariate study | | | Multivariate study | | |
|---|---|---|---|---|---|---|---|
| | RL | RR | 95% CI | P | RR | 95% CI | P |
| P27 | ≤5% | 1.6491 | 1.0438–2.6053 | 0.0320 | 1.7811 | 1.1210–2.8300 | 0.0145 |
| MIB1 | ≤20% | 1.7554 | 1.0931–2.8190 | 0.0199 | 1.5514 | 0.9420–2.5550 | 0.0845 |
| IPI | | | | 0.0004 | | | 0.0010 |
| IPI(2) | IPI(0–1) | 2.7338 | 1.2761–5.8568 | 0.0097 | 2.7513 | 1.2796–5.9156 | 0.0096 |
| IPI(3) | IPI(0–1) | 3.5224 | 1.6555–7.4945 | 0.0011 | 3.6285 | 1.7024–7.7337 | 0.0008 |
| IPI(4–5) | IPI(0–1) | 4.8971 | 2.3176–10.3474 | 0.0000 | 4.5606 | 2.1337–9.7479 | 0.0001 |
| Disease-free survival | | | | | | | |
| P27 | ≤5% | 2.2732 | 1.0334–5.0005 | 0.0409 | 2.2899 | 1.0349–5.0669 | 0.0409 |
| MIB1 | ≤20% | 1.1531 | 0.5180–2.5668 | 0.7271 | 1.4448 | 0.6060–3.4446 | 0.4065 |
| IPI | | | | 0.1475 | | | 0.1148 |
| IPI(2) | IPI(0–1) | 2.6184 | 0.9204–7.4491 | 0.0712 | 2.6280 | 0.9133–7.5620 | 0.0732 |
| IPI(3) | IPI(0–1) | 3.1793 | 1.1018–9.1740 | 0.0324 | 3.3917 | 1.1662–9.8646 | 0.0250 |
| IPI(4–5) | IPI(0–1) | 1.4543 | 0.2931–7.2165 | 0.6468 | 1.1496 | 0.2169–6.0924 | 0.8698 |

Chi-square: 28.949, $P < 0.0001$ for overall survival; Chi-square: 10.736, $P = 0.0569$ for disease-free survival. RL: reference level; RR: risk ratio; CI: confidence interval.

© 1999 Cancer Research Campaign tissues between p27$^{KIP1}$ and MIB1 expression is lacking. This anomalous p27$^{KIP1}$ expression was also associated with an adverse clinical outcome in univariate as well as multivariate analysis.

This finding contradicts observations in reactive lymphoid tissue, where p27$^{KIP1}$ expression is always associated with quiescent status, as shown by immunohistochemistry of reactive lymph nodes and tonsils, flow cytometry and Western blot studies of phytohaemaglutinin-stimulated peripheral blood lymphocytes. These show that the p27$^{KIP1}$ protein is mainly found in MIB-1 negative quiescent lymphocytes, its expression being down-regulated along cell cycle progression (Sánchez-Beato et al, 1997). Thus benign centroblasts, the proliferating B-cell subset of the germinal centre and the supposed normal counterpart of most DLBCL, usually show a MIB1+, p27– phenotype, which is lost to a certain extent in DLBCL. This had already been noticed in a previous study of NHL by our group, in which the existence of a small group of cases of Burkitt's lymphoma and DLBCL with this anomalous nuclear p27$^{KIP1}$ accumulation was described (Sánchez-Beato et al, 1997); these results were confirmed by further studies by Quintanilla-Martínez et al (1998) and Erlanson et al (1998). A more detailed study was therefore performed. This analysed a larger series of cases using a more sensitive technique and quantified the results with a more analytical procedure, including statistical analysis of the relationship between p27$^{KIP1}$, MIB1 and survival probability. This made it possible to confirm and expand earlier observations, indicating that a gradient of p27$^{KIP1}$ expression may be seen in DLBCL. Contrary to what was expected on the basis of the findings in non-tumoural tissues and other tumours, p27$^{KIP1}$ expression appears to be associated with an unfavourable clinical course, thereby supporting the hypothesis that this abnormal accumulation of p27$^{KIP1}$ protein is deregulated (Sgambato et al, 1997). To exclude the possibility that the adverse effect of p27$^{KIP1}$ expression on survival could be attributed exclusively to the small group of cases with very high p27$^{KIP1}$ expression, we repeated the analysis excluding the cases with values of p27 > 25%, the same findings being repeated in this group (data not shown). This same series of patients was the subject of a recent report showing that high growth fraction is an adverse prognostic factor in multivariate analysis (Sánchez et al, 1997). Now, and once p27$^{KIP1}$ is included in the analysis, the predictive value of MIB1 is lost in favour of that of p27$^{KIP1}$.

The techniques used here for the quantitative analysis of p27$^{KIP1}$ and MIB1 reflect the difficulties arising in estimating these in DLBCL. As is the case for most reactive T-cells, endothelial cells and histiocytes are p27$^{KIP1}$-positive. We therefore decided to count the cells manually, thereby selecting tumoural cells exclusively on the basis of their morphology. This made it possible to recognize and estimate the presence of a minority p27-positive tumoural cell subpopulation in a significant group of DLBCL. This finding was also described in the other analysis published to date of p27$^{KIP1}$ expression in DLBCL, thereby underlining the consistency of these techniques and the reproducibility of the results described. Thus, the studies of Quintanilla-Martínez et al (1998) and Erlanson et al (1998) both describe the presence of anomalous p27$^{KIP1}$ expression in a subset of DLBCL cases, together with an associated loss of the normal p27/MIB1 ratio found in normal cells.

Nevertheless, software specifically designed for the recognition of the growth fraction of tumours was used for MIB1 analysis, since most positive cells in DLBCL are members of the tumoural cell population, which makes it possible to use an image analysis program. A relatively low percentage of proliferating cells was found in this study, depending of the characteristics of the software used, and this must be taken into consideration when comparing these values with those corresponding to other series.

Although the loss of the inverse relationship between p27$^{KIP1}$ and MIB-1 expression has been described previously in different studies of breast and colorectal cancer specimens and derived cell lines (Catzavelos et al, 1997; Fredersdorf et al, 1997; Lodu et al, 1997; Sgambato et al, 1997), the existence of an adverse clinical significance for increased expression of p27$^{KIP1}$ in a specific tumoural type has only been rarely noticed. Indeed, earlier reports emphasized that a more favourable clinical outcome is usually associated with a high level of p27$^{KIP1}$ expression, as seen in non-small-cell lung cancer (Esposito et al, 1997), breast cancer (Catzavelos et al, 1997; Porter et al, 1997), colorectal cancer (Loda et al, 1997). A recent report by Erlanson et al (1998) found that, when all histological types of NHL are taken into consideration, high p27$^{KIP1}$ values are associated with longer survival probabilities, and therefore probably reflect the fact that the study simultaneously includes different entities, while it does not analyse the specific meaning of p27$^{KIP1}$ expression in each histological type. Nevertheless, when considering a specific entity like B-CLL, a recent analysis by Vrhovac et al (1998) illustrates the same phenomenon found here, i.e. high p27$^{KIP1}$ levels associated with a poorer overall prognosis, which appeared to be related to the association between high p27$^{KIP1}$ and apoptosis resistance. These results overlap those of other reports in epithelial tumours, in which an anomalous deregulated p27$^{KIP1}$ expression in some tumoural cell types has been described (Sgambato et al, 1997; Yatube et al, 1998).

It has been proposed that p27$^{KIP1}$ overexpression is involved in the drug resistance displayed by solid tumours, and the suggestion has even been made that tumour-targeted p27$^{KIP1}$ antagonists could be used in conjunction with conventional anticancer therapy (Croix et al, 1996). In B-CLL it has been shown that an increased p27$^{KIP1}$ level protects cells against apoptosis, this being reverted after fludarabin exposure (Vrhorac et al, 1998). These experimental data could explain the shorter survival in the patients analysed here.

This abnormally accumulated p27$^{KIP1}$ protein seems to lack the capacity for inhibiting Cyclin E–CDK2 complexes, since these aggressive B-cell lymphomas have a high proliferative index and an unfavourable clinical outcome. This situation has also been observed in human breast cancer cell lines, where an increase in p27$^{KIP1}$ expression was paralleled by an increase in the level of Cyclin D1 and Cyclin E. As p27$^{KIP1}$ expression is mainly regulated at a post-transcriptional level, it has therefore been suggested that there is a homeostatic mechanism for the up-regulation of p27$^{KIP1}$, induced by overexpression of Cyclin D1 or Cyclin E (Sgambato et al, 1997). Additional works by Blain et al (1997), Cheng et al (1998), Reynisdottir and Massague (1997) and Soos et al (1996) have shown that a high level of p27$^{KIP1}$ expression may be found in growing cells, confirming that this negative regulator of the cell cycle is kept inactive by adhesion to Cyclin D–CDK4 complexes. This could also be an hypothetical explanation for the anomalous p27$^{KIP1}$ expression in this group of DLBCL.

It is still not known if the adverse clinical meaning of p27$^{KIP1}$ expression in DLBCL is the direct consequence of the accumulation of this protein, or whether it is merely a reflection of a deeper alteration of cell cycle regulator genes leading to this anomalous p27$^{KIP1}$ expression.

© 1999 Cancer Research Campaign

### ACKNOWLEDGEMENTS

We would like to thank Isabel Gálvez-Torija and Alicia Muñoz for their excellent technical assistance. We extend our appreciation to Professor AJ Sáez-Castillo of Jaén University for his contributions in helping to set up statistical analyses. Dr Elías Campo kindly contributed to the manuscript by discussing the data and the text of the article. This work was supported by grants from the Fondo de Investigaciones Sanitarias and the Fundación Areces, Spain.

### REFERENCES

Bani MR, Rak J, Florenes VA, Ben-David Y and Kerbel RS (1997) Overexpression of $p27^{KIP1}$ is associated with enhanced survival under three-dimensional growth conditions during melanoma progression. *Proc Am Assoc Cancer Res* **38**: 496

Blain SW, Montalvo E and Massague J (1997) Differential interaction of the cyclin-dependent kinase (CDK) inhibitor $p27^{KIP1}$ with cyclin A-CDK2 and cyclin D2-CDK4. *J Biol Chem* **41**: 25863–25872

Catzavelos C, Bhattacharva N, Ung Y, Wilson J, Roncari L, Sandhu C, Shaw P, Yeger H, Morava-Protzner I, Kapusta L, Franssen E, Pritchard K and Slingerland J (1997) Decreased levels of the cell cycle inhibitor $p27^{KIP1}$ protein: prognostic implications in primary breast cancer. *Nat Med* **3**: 227–230

Chan FKM, Zhang J, Cheng L, Shapiro DN and Winoto A (1995) Identification of human and mouse p19, a novel CDK4 and CDK6 inhibitor with homology to p16INK4. *Mol Cell Biol* **15**: 2682–2688

Cheng M, Sexl V, Sherr CJ and Rousell MF (1998) Assembly of cyclin D-dependent kinase and titration of p27KIP1 regulated by mitogen-activated protein kinase (MEK1). *Proc Natl Acad Sci USA* **95**: 1092–1096

Coats S, Flanagan WM, Nourse J and Roberts JM (1996) Requirement of $p27^{KIP1}$ for restriction point control of the fibroblasts cell cycle. *Science* **2**: 877–880.

Cox DR (1972) Regression models and life tables. *J R Stat Soc* (B) **34**: 187–220.

Croix BS, Florenes VA, Rak JW, Flanagan M, Bhattacharya N, Slingerland JM and Kerbel RS (1996) Impact of the cyclin-dependent kinase inhibitor $p27^{KIP1}$ on resistance of tumor cells to anticancer agents. *Nat Med* **2**: 1204–1210.

El-Deiry WS, Tokino T, Veculescu VE, Levy DB, Parsons R, Trent JM, Lin D, Mercer E, Kinzler KW and Vogelstein B (1993) WAF1, a potential mediator of p53 tumor suppression. *Cell* **75**: 817–825

Erlanson M, Portin C, Linderholm B, Lindh J, Roos G and Landberg G (1998) Expression of cyclin E and the cyclin-dependent kinase inhibitor p27 in malignant lymphomas; prognostic implications. *Blood* **92**: 770–777

Esposito V, Baldi A, De Luca A, Groger AM, Loda M, Giordano GG, Caputi M, Baldi F, Pagano M and Giordano A (1997) Prognostic role of the cyclin-dependent kinase inhibitor $p27^{KIP1}$ in non-small-cell lung cancer. *Cancer Res* **15**: 3381–3385

Fang F, Orend G, Watanabe N, Hunter T and Ruoslathi E (1996) Dependence of cyclin E–CDK2 kinase activity on cell anchorage. *Science* **271**: 499–502

Fero ML, Rivkin M, Tasch M, Porter P, Carow CE, Firpo E, Polyak K, Tsai L-H, Broudy V, Perlmutter, Kaushansky K and Roberts JM (1996) A syndrome of multiorgan hyperplasia with features of gigantism, tumorigenesis, and female sterility in $p27^{KIP1}$-deficient mice. *Cell* **85**: 733–744

Fredersdorf S, Burns J, Milne AM, Packham G, Fallis L, Gillett CE, Royds JA, Peston D, Hall PA, Hanby AM, Barnes DM, Shousha S, O'Hare MJ and Lu X (1997) High level expression of $p27^{KIP1}$ and cylin D1 in some human breast cancer cells: Inverse correlation between the expression of $p27^{KIP1}$ and degree of malignancy in human breast and colorectal cancers. *Proc Natl Acad Sci USA* **94**: 6380–6385

Gascoyne RD, Adomat SA, Krajewski S, Krajewska M, Horsman DE, Tolcher AW, O'Reilly SE, Hoskins P, Coldman AJ, Reed JC and Connors JM (1997) Prognostic significance of Bcl-2 protein expression and *bcl-2* gene rearrangement in diffuse aggressive non-Hodgkin's lymphoma. *Blood* **90**: 244–251

Grogan TM, Lippman SM, Spier CM, Slymen DJ, Rybski JA, Rangel CS, Richter LC and Miller TP (1988) Independent prognostic significance of a nuclear proliferation antigen in diffuse large cell lymphomas as determined by the monoclonal antibody Ki-67. *Blood* **71**: 1157–1160

Guan K-L, Jenkins CW, Li Y, Nichols MA, Wu X, O'Keefe CL, Matera AG and Xiong Y (1994) Growth suppression by p18, a $p16^{INK4/MTS1}$ and $p14^{INK4/MTS2}$-related CDK6 inhibitor, correlates with wild-type pRb function. *Genes Dev* **8**: 2939–2952

Hannon GJ and Beach D (1994) $p15^{INK4B}$ is a potential effector of TGF-β induced cell cycle arrest. *Nature* **371**: 257–261

Harper JW, Adami GY, Wei N, Keyomarsi K and Elledge SJ (1993) The p21 Cdk-interacting protein Cip1 is a potent inhibitor of G1 cyclin-dependent. *Cell* **75**: 805–816

Harris NL, Jaffe ES, Stein H, Banks PM, Chan JK, Cleary ML, Delsol G, De Wolf-Peeters C, Falini B and Gatter KC (1994) A revised European–American classification of lymphoid neoplasm: A proposal from the International Lymphoma Study Group. *Blood* **84**: 1361–1392

Hengst L and Reed SI (1996) Translational control of $p27^{KIP1}$ accumulation during the cell cycle. *Science* **271**: 1861–1864

Hermine O, Haioun C, Lepage E, d'Agay MF, Briere J, Lavignac C, Fillet G, Salles G, Marolleau JP, Diebold J, Reyas F and Gaulard P, for the Groupe d'Etude des Lymphomes de l'Adulte (GELA) (1996) Prognostic significance of bcl-2 protein expression in aggressive non-Hodgkin's lymphoma. *Blood* **87**: 265–272

Hill ME, MacLennan KA, Cunningham DC,Vaughan Hudson B, Burke M, Clarke P, Di Stefano F, Anderson L, Vaughan Hudson G, Mason D, Selby P and Linch DC (1996) Prognostic significance of Bcl-2 expression and bcl-2 major breakpoint region rearrangement in diffuse large non-Hodgkin's lymphoma: a British national lymphoma investigation study. *Blood* **88**: 1046–1051

Kaplan EL and Meier P (1958) Non-parametric estimation for incomplete observations. *J Am Stat Assoc* **53**: 457–481

Kato J, Matsuoka M, Polyak K, Massague J and Sherr CJ (1994) Cyclic AMP-induced G1 phase arrest mediated by an inhibitor ($p27^{KIP1}$) of cyclin-dependent kinase 4 activation. *Cell* **79**: 487–496

Kawamata N, Morosetti R, Miller CW, Park D, Spirin KS, Nakamaki T, Takeuchi S, Hatta Y, Simpson J, Wilczynski S, Lee YY, Bartran CR and Koeffler HP (1995) Molecular analysis of the cyclin-dependent kinase inhibitor gene $p27^{KIP1}$ in human malignancies. *Cancer Res* **55**: 2266–2269

Kiyokawa H, Kineman RD, Manova-Todorova KO, Soares VC, Hoffman ES, no M, Khanam D, Hayday AC, Frohman LA and Koff A (1996) Enhanced growth of mice lacking the cyclin-dependent kinase inhibitor function of $p27^{KIP1}$. *Cell* **85**: 721–732

Kramer MHH, Hermans J, Parker J, Krol AD, Kluin-Nelemans JC, Haak HL, van Groningen K, van Krieken JH, de Jong D and Kluin PM (1996) Clinical significance of bc12 and p53 protein expression in diffuse large B-cell lymphoma: a population-based study. *J Clin Oncol* **14**: 2131–2138

Lee MH, Reynisdóttir I and Massagué J (1995) Cloning of $p57^{KIP2}$, a cyclin-dependent kinase inhibitor with unique domain structure and tissue distribution. *Genes Dev* **9**: 639–649

Loda M, Cukor B, Tam SW, Lavin P, Fiorentino M, Draetta GF, Jessup M and Pagano M (1997) Increased proteasoma-dependent degradation of the cyclin-dependent kinase inhibitor p27 in aggressive colorectal carcinomas. *Nat Med* **3**: 231–235

Mantel C, Luo Z, Canfield J, Braun S, Deng C and Broxmeyer HE (1996) Involvement of $p21^{CIP1}$ and $p27^{KIP}$ in the molecular mechanism of Steel Factor-induced proliferative synergy in vitro and of $p21^{CIP1}$ in the maintenance of stem/progenitor cells in vivo. *Blood* **88**: 3710–3719

Mantel N (1966) Evaluation of survival and two new rank order statistics arising in its consideration. *Cancer Chemother Rep* **55**: 163–170

Martin AR, Weisenburger DD, Chan WC, Ruby EI, Anderson JR, Vose JM, Bierman PJ, Bast MA, Daley DT and Armitage JO (1995) Prognostic value of cellular proliferation and histologic grade in follicular lymphoma. *Blood* **85**: 3671–3678

Matsuoka S, Edwars M, Bai C, Parker S, Zhang P, Baldin A, Harper JW and Elledge SJ (1995) $p57^{KIP2}$, a structurally distinct member of the Cip1 CDK inhibitor family, is a candidate tumor suppressor gene. *Genes Dev* **9**: 650–662

Miller TP, Grogan TM, Dahlberg S, Spier CM, Braziel RM Banks PM, Foucar K, Kjeldsberg CR, Levy N, Nathwani BN, Schnitzer B, Tubbs RR, Gaynor ER and Fisher RI (1994) Prognostic significance of the Ki-67-associated proliferative antigen in aggressive non-Hodgkin's lymphomas: a prospective Southwest Oncology Group Trial. *Blood* **83**: 1460–1466

Morosetti R, Kawamata N, Gombart AF, Miller CW, Hatta Y, Hirama T, Said JW, Tomonaga M and Koeffler HP (1995) Alterations of the $p27^{KIP1}$ gene in non-Hodgkin's lymphomas and adult T-cell leukaemia/lymphoma. *Blood* **86**: 1924–1930

Nakayama K, Ishida N, Shirane M, Inomata A, Inoue T, Shishido N, Horii I, Loh DY and Nakayama K-I (1996) Mice lacking $p27^{KIP1}$ display increased body size, multiple organ hyperplasia, retinal dysplasia, and pituitary tumors. *Cell* **85**: 707–720

Nourse J, Firpo E, Flanagan WM, Coats S, Polyak K, Lee M-H, Massague J, Crabtree GR and Roberts JM (1994) Interleukin-2-mediated elimination of the $p27^{KIP1}$ cyclin-dependent kinase inhibitor prevented by rapamycin. *Nature* **372**: 570–573

Pietenpol JA, Bohlander SK, Sato y, Papadopoulos N, Liu B, Friedman C, Trask BJ, Roberts JM, Kinzler KW, Rowly JD and Vogelstein B (1995) Aassignment of

© 1999 Cancer Research Campaign the human p27[KIP1] gene to 12p13 and its analysis in leukemias. *Cancer Res* **55**: 1206–1210.

Piris MA, Pezella F, Martinez-Montero JC,Orradre JL, Villuendas R, Sanchez-Beato M, Cuena R, Cruz MA, Martinez B, Garrido MC, Gatter K, Aiello A, Delia D, Giardini R and Rilke F (1994) p53 and bcl-2 expression in high-grade B-cell lymphomas: correlation with survival time. *Br J Cancer* **69**: 337–341

Polyak K, Lee M-H, Erdjument-Bromage H, Koff A, Roberts JM, Tempst P and Massague J (1994) Cloning of p27[KIP1], a cylin-dependent kinase inhibitor and a potential mediator of extracellular antimitogenic signals. *Cell* **78**: 59–66

Porter P, Malone K, Heagerty P, Alexander G, Gatti L, Firpo E, Daling J and Roberts J (1997) Expression of cell-cycle regulators p27[KIP1] and cyclin E, alone and in combination, correlate with survival in young breast cancer patients. *Nat Med* **3**: 222–225

Quintanilla-Martinez L, Thieblemont C, Fend F, Kumar S, Pinyol M, Campo E, Jaffe E and Raffeld M (1998) Mantle cell lymphomas lack expression of p27KIP1 a cyclin-dependent kinase inhibitor. Am *J Pathol* **153**: 175–182

Reynisdottir I and Massague J (1997) The subcellular locations of p15INK4b and p27[KIP1] co-ordinate their inhibitory interactions with CDK4 and CDK2. *Genes Dev* **11**: 492–503

Sánchez E, Chacon I, Plaza MM, Muñoz E, Cruz MA, Martinez B, Lopez L, Martinez-Montero JC, Orrader JL, Saez AI, Garcia JF and Piris MA (1998) Clinical outcome in diffuse large B-cell lymphoma is dependent on the relationship between different cell cycle regulator proteins. *J Clin Oncol* **16**: 1931–1939

Sánchez-Beato M, Sáez AI, Martínez-Montero JC, Mateo MS, Sánchez-Verde L, Villuendas R, Troncone G and Piris MA (1997) Cyclin-dependent kinase inhibitor p27[KIP1] in lymphoid tissue: p27[KIP1] expression is inversely proportional to the proliferative index. *Am J Pathol* **151**: 151–160

Serrano M, Hannon GJ and Beach D (1993) A new regulatory motif in cell cycle control causing specific inhibition of cyclin D/CDK4. *Nature* **366**: 704–707

Sgambato A, Zhang Y, Arber N, Hibshoosh H, Doki Y, Ciaparrone M, Santella RM, Cittadini A and Weinstein IB (1997) Deregulated expression of p27[KIP1] in Human Breast Cancers. *Clinical Cancer Res* **3**: 1879–1887

Shiohara M, El-Deiry WS, Wada M, Nakamaki T, Takeuchi S, Yang R, Chen DL, Vogelstein B and Koeffler HP (1994) Absence of Waf1 mutations in a variety of human malignancies. *Blood* **84**: 3781–3784

Slymen DJ, Miller TP, Lippman SM,Spier CM, Kerrigan DP, Rybski JA, Rangel CS, Richter LC and Grogan TM (1990) Immunobiologic factors predictive of clinical outcome in diffuse large-cell lymphoma. *J Clin Oncol* **8**: 986–993

Soos TJ, Kiyokawa H, Yan JS, Rubin MS, Giordano A, DeBlasio A, Bottega S, Wong B, Mendelsohn J and Koff A (1996) Formation of p27–CDK complexes during the human mitotic cell cycle. *Cell Growth Differ* **7**: 135–146

The International Non-Hodgkin's Lymphoma Prognostic Project (1993) A predictive model for aggressive non-Hodgkin's lymphoma. *N Engl J Med* **329**: 987–994

Toyoshima H and Hunter T (1994) p27[KIP1], a novel inhibitor of G1 cyclin–CDK protein kinase activity, is related to p21. *Cell* **78**: 67–74

Vrhorac R, Delmer A, Tang R, Marie JP, Zittoun R and Ajchenbaum-Cymbalista F (1998) Prognostic significance of the cell cycle inhibitor p27[KIP1] in chronic B-cell lymphocytic leukemia. *Blood* **91**: 4694–4700

Wang X, Gorospe M, Huang Y and Holbrook NJ (1997) p27[KIP1] overexpression causes apoptoic death of mammalian cells. *Oncogene* **15**: 2991–29978

Watanabe H, Fukuchi K, Takagi Y, Tomoyasu S, Tsuruoka N and Gomi K (1995) Molecular analysis of the Cip1/Waf1 (p21) gene in diverse types of human tumors. *Biochim Biophys Acta* **1263**: 275–278

Yamanaka N, Harabuchi Y and Kataura A (1992) The prognostic value of Ki-67 antigen in non-Hodgkin's lymphoma of Waldeyer ring and the nasal cavity. *Cancer* **70**: 2342–2349

Yatabe Y, Masuda A, Koshikawa T, Nakamura S, Kuroishi T, Osada H, Takahashi T, Mitsudomi T and Takahashi T (1998) P27[KIP1] in human lung cancers: differential changes in small-cell and non-small-cell carcinomas. *Cancer Res* **58**: 1042–1047

 *© 1999 Cancer Research Campaign*
